# Under the Radar: A Case Report of a Missed Aortoenteric Fistula

**DOI:** 10.5811/cpcem.2021.4.51791

**Published:** 2021-07-27

**Authors:** Blake Briggs, David Manthey

**Affiliations:** * University of South Alabama, Department of Emergency Medicine, Mobile, Alabama; † Wake Forest University, Department of Emergency Medicine, Winston-Salem, North Carolina

**Keywords:** Vascular surgery, aortoenteric fistula, radiology, gastrointestinal bleeding

## Abstract

**Introduction:**

An aortoenteric fistula (AEF) is an abnormal connection between the aorta and the gastrointestinal tract that develops due to a pathologic cause. It is a rare, but life-threatening, cause of gastrointestinal (GI) bleeding. Although no single imaging modality exists that definitively diagnoses AEF, computed tomography angiography (CTA) of the abdomen and pelvis is the preferred initial test due to widespread availability and efficiency.

**Case Report:**

Many deaths occur before the diagnosis is made or prior to surgical intervention. We describe a case of a patient with a history of aortic graft repair who presented with active GI bleeding.

**Conclusion:**

Although CTA can make the diagnosis of AEF, it cannot adequately rule it out. In patients with significant GI bleeding and prior history of aortic surgery, vascular surgery should be consulted early on, even if CTA is equivocal.

## INTRODUCTION

An aortoenteric fistula (AEF) is an abnormal connection that forms between the aorta and the gastrointestinal tract due to pathologic cause. It is a rare but life-threatening condition with an annual incidence of 0.007 per million. Primary causes are due to compression of an abdominal aortic aneurysm (AAA) against gastrointestinal (GI) structures. In these cases, there is usually some type of inflammation affecting the aorta, whether it be septic aortitis from bacteremia, cancer, inflammatory bowel disease, peptic ulcer, radiation, perforating biliary stones, or autoimmune disease.^1^ Primary AEFs have an incidence of 0.04–0.07%. The most affected portion of bowel in AEF is between the infrarenal aorta and the third and fourth portion of the duodenum.^2^

Secondary etiology is due to erosion of an aortic prosthetic graft after an open repair into the surrounding GI structures.^3^ The most frequently affected portion of the bowel is the third portion of the duodenum, likely due to its retroperitoneal fixation and proximity to aorta.^4^ Secondary aortoenteric fistulas (SAEF) are far more common than primary.^5^ The overall incidence of SAEF has been measured at 0.36–1.6%. They are exceedingly rare after endovascular aneurysm repair.

Gastrointestinal bleeding is the most common initial presentation, occurring in 90% of patients. Abdominal pain is only present in 28%, and fever is present in up to 25%.^4^ The classically taught triad of GI bleeding, abdominal pain, and a palpable mass, however, is seen in only 6–12% of patients. One study demonstrated that only 29% of patients arrived with massive hemorrhage.^6^ Consequently, diagnosis is not easy due to its rarity and varied presentation, requiring astute clinical judgment. Early diagnosis of SAEF often relies upon recognition of typical “herald bleed,” which is an episode of self-limited bleeding that precedes catastrophic hemorrhage.

Although no single imaging modality definitively diagnoses SAEF, computed tomography angiography (CTA) of the abdomen and pelvis is the preferred initial test due to widespread availability and efficiency. However, CTA has been found to range from 40–90% sensitive and 33–100% specific.^7^ This is likely due to the aspects surrounding AEF. Inflammation often precludes proper viewing of the affected area, the graft bleeding can often be a small site, and the bleeding might be intermittent and not occurring at the time of radiographic imaging.^8^–^10^

We discuss a case where the abdominal CTA was equivocal for suspected SAEF, leading to delayed diagnosis and mortality. This case report emphasizes the importance of maintaining high clinical suspicion for a rare but deadly etiology of GI bleeding, and to understand the limitations of studies used to identify it. Without treatment, AEF is universally fatal.

## CASE REPORT

A 74-year-old female presented from her skilled nursing facility after development of dark tarry stool from her ostomy and one episode of dark red hematemesis about two hours prior. She denied any abdominal pain. On arrival she was found to be hypotensive with mean arterial pressure of 50 millimeters of mercury and heart rate in the 120s. The patient had a complicated past medical history, including autoimmune hepatitis, AAA mesh repair in 2015 complicated by pulseless electrical activity arrest, fourth thoracic vertebrae infarct resulting in paraplegia, and neurogenic bladder requiring a chronic indwelling catheter. She also had a left hemicolectomy secondary to ischemic colitis, requiring an ostomy. The patient took 81 milligrams aspirin daily, and otherwise was not on any blood thinners. She had no history of peptic ulcer disease and was not on chronic nonsteroidal anti-inflammatory medications.

**Image 1:**
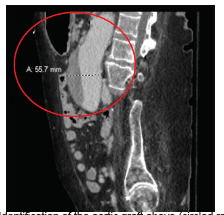
Identification of the aortic graft above (circled red), demonstrating no contrast extravasation or fistula.

**Image 2:**
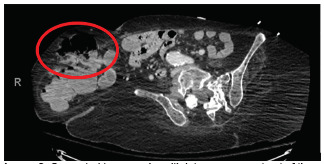
Computed tomography with intravenous contrast of the abdomen and pelvis. No active extravasation was found, and in the circled area above in the parastomal hernia (large red circle).

Immediately on arrival the patient was given one liter crystalloid with no improvement; therefore, one unit whole blood was given, followed by multiple units of emergency release packed red blood cells. While in the emergency department (ED), the patient’s ostomy bag was changed multiple times due to it being filled with dark red bloody stool. A CT abdomen and pelvis with intravenous (IV) contrast was performed but did not identify any obvious source of acute GI bleeding or acute pathology. Clear identification of the aortic graft occurred but showed no contrast extravasation or fistula (Image 1 and 2).

**CPC-EM Capsule**

What do we already know about this clinical entity?

*An aortoenteric fistula (AEF) is a rare cause of gastrointestinal (GI) bleeding most often diagnosed with computed tomography angiography (CTA)*.

What makes this presentation of disease reportable?

*We describe a case of a patient with a history of aortic graft repair presenting with active GI bleeding whose CTA was equivocal, leading to delayed diagnosis*.

What is the major learning point?

*In patients with significant GI bleeding and prior history of aortic surgery, vascular surgery should be consulted early on, even if CTA is equivocal*.

How might this improve emergency medicine practice?

*Early consultation of vascular surgery is critical as mortality approaches 100% if AEF is not treated in a timely fashion*.

**Image 3:**
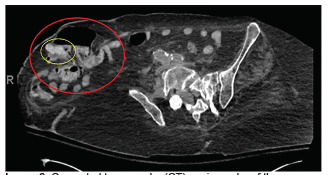
Computed tomography (CT) angiography of the abdomen and pelvis. This scan was performed hours after the CT (Image 2). It was found that inside the parastomal hernia (large red circle), contrast had appeared in this hernia, which was not previously visible on prior CT (circled yellow).

After consultation with gastroenterology, the patient was admitted and rapidly transferred to the medical intensive care unit (ICU). By that time, she continued to have intermittent output from her ostomy. Shortly after admission she was administered vasopressor; her mental status declined, and she was intubated. The patient became too unstable for esophagogastroduoden-oscopy (EGD), and so CTA was performed. The CTA showed no active extravasation, but there was contrast within the distal colon and within parastomal hernia from the original contrasted study on pre-contrast images of the CTA (Image 3). Interventional radiology was consulted, but they deferred arteriography given lack of active extravasation on CTA.

Hours later, the patient had increased pressor requirements, and there was renewed concern for active bleeding. While preparing for EGD, the patient’s metabolic acidosis worsened. Lactate increased to 12.5 millimoles per liter (mmol/L) (reference range 2–4 mmol/L), white blood cell count 33 (4.5–11 × 10^9^/L), and arterial blood pH 7.24. Vascular surgery consult voiced concerned for AEF. By this time the patient had rapidly declined in her course of illness and surgery was no longer a viable option. Due to decompensation with multisystem organ failure, further discussions were held with the patient’s two daughters, and the decision was made to move her to comfort care.

## DISCUSSION

This case highlights two important concepts germane to emergency physicians: The first is to maintain a high level of suspicion for life-threatening diseases, especially rare ones; and the second is to understand the difference between the timing of a CT with contrast and one timed as an aortogram.

Secondary AEF is difficult to diagnose for several reasons. It is a rare disease that can occur with or without aortic aneurysmal repair. It may not present as GI bleeding, but instead with hypotension and a fever mimicking septic shock. Myriad causes may be blamed for the apparent GI bleeding, especially if it is self-limited. We would argue that any patient with a history of an open aortic aneurysmal repair with GI bleeding should be considered an SAEF until proven otherwise. Given the often-vague presentation yet high propensity of morbidity and mortality in AEF, consulting vascular surgery early is the right decision.

In this case, there was never definitive radiographic proof (eg, active extravasation, periaortic edema, graft thrombosis, or thickening and close proximity of the graft to the bowel) that an AEF was present. The first CT ordered was with contrast. This study, in comparison to the gold standard of CT angiography, is not timed to follow the contrast through the vasculature, and thus may not identify active extravasation. Unfortunately, in this case, the CT with IV contrast added a false sense of security among the ED, ICU, GI, and interventional radiology teams.

Computed tomography angiography of the abdomen and pelvis, with and without contrast, is the preferred initial test. Traditional CT with IV contrast does not have adequate sensitivity and specificity for detecting aortic injuries, and its inability to perform accurate arterial-phase scanning followed by detailed 1-millimeter (mm) collimation makes it an inferior first-line test when concerned about AEF. However, CTA is not without limitations. It has been found to range from 40–90% sensitivity and 33–100% specificity. Typical features on CTA that suggest AEF include ectopic gas adjacent to, or within, the aorta, focal bowel wall thickening, discontinuity of the aortic wall, and active extravasation of contrast into the bowel lumen. Additionally, the fat plane between the aorta and bowel is often obliterated along the affected segment. In most cases the site of the fistula is between the proximal suture line and the duodenum.

It must be emphasized that CTA of the abdomen and pelvis must be performed properly to not limit its sensitivity and specificity.^13^ All aorta evaluations should occur on 16- or 64-detector CT scanners. Initially, unenhanced scanning should occur, followed by arterial-phase CT performed with bolus contrast tracking and 1-mm collimation. Importantly, there must be an 80-second delay before imaging of the abdomen. This delayed imaging should be performed to detect endoluminal leakage when an aortic graft or stent is present. Oral contrast should never be used, as it can obscure subtle extravasation from the aorta into the bowel lumen.^12^ A positive, as well as an indeterminate or equivocal CTA in a patient with active or intermittent GI bleeding, should prompt emergent vascular surgery consultation. Traditional CT with IV contrast has very limited sensitivity and specificity and should not be relied upon to rule out AEF.

The CTA in this case demonstrated contrast in the distal colon and parastomal hernia on pre-contrast images, which implied contrast from the prior CT of the abdomen and pelvis performed hours earlier had moved from the vasculature into the GI tract. This finding is expected in a patient with a GI bleed but does not indicate the origin of the contrast-containing blood. Deferring arteriography due to a lack of active extravasation may be argued from a standpoint of efficacy of the study.

Vascular surgery was consulted very late in the patient’s disease course – too late to offer surgical intervention. Without surgical intervention, AEFs are virtually 100% fatal.^12^ When surgery was performed, one study found mortality to be 36%. Most deaths occurred before the diagnosis was made or prior to surgical intervention.^12^ The risk of mortality of this patient who presented in extremis with need for blood products, however, was very high, especially given her age and other underlying medical conditions.

As is often the case in patients with GI bleeding, gastroenterology was primarily consulted instead of vascular surgery. However, endoscopy is not the preferred evaluation or management of AEFs. The sensitivity of endoscopy for SAEF is only 50%.^13^ Interestingly, peptic ulcers were found three times more often in association with AAA than in the general population.^14^ Even more concerning, a normal endoscopy or one positive for peptic ulcer disease without active bleeding does not definitively rule out AEF.^15^

## CONCLUSION

Aortoenteric fistula remains a rare but deadly cause of GI bleeding. Without treatment, AEF is universally fatal. Any patient with a history of aortic surgery and GI bleeding should be considered to have an AEF until proven otherwise, and CTA should be used early on. Computed tomography angiography can rule in the diagnosis of AEF quite well, and is considered the first-line test, but it cannot adequately rule it out.^8^ In patients with GI bleeding and prior history of aortic surgery, vascular surgery should be consulted early on, even if CTA is equivocal.
